# ChatGPT: immutable insertion in health research and researchers’ lives

**DOI:** 10.31744/einstein_journal/2024CE0752

**Published:** 2024-02-29

**Authors:** Aléxia Gabriela da Silva Vieira, Humberto Saconato, Raquel Afonso Caserta Eid, Ricardo Kenji Nawa

**Affiliations:** 1 Hospital Israelita Albert Einstein São Paulo SP Brazil Hospital Israelita Albert Einstein, São Paulo, SP, Brazil.; 2 Universidade Federal de São Paulo São Paulo SP Brazil Universidade Federal de São Paulo, São Paulo, SP, Brazil.

Dear Editor,

Scientific advancements driven by an increased understanding of big data, patient preferences, and high-quality research have revealed the need for rapid responses.^(1)^ In this scenario, the use of artificial intelligence (AI), which enables the possibility of obtaining full texts, which mimic human language patterns, from a short sentence, with the adoption of AI tools such as Chat generative Pre-trained Transformer-ChatGPT (Open AI, San Francisco, CA, USA), is attractive.^(2)^ A growing number of researchers are evaluating the potential of ChatGPT to aid in diagnostic decisions, report generation, and patient education through prompts; however, other important facets of AI use need to be explored.^(3,4)^

The super support of ChatGPT in the field of research can assist in or perform all the work related to writing and summarizing scientific texts, search for and show outputs to answer questions faster (though not as accurately), process large volumes of data, and facilitate literature reviews, thus saving time.^(5-7)^ In addition, the constant learning algorithm allows the user to request ideas. Optimization of textual synthesis of ‘health topics’ can guarantee more time for critical evaluation of the content and reflections on the applicability of the content for different scenarios.^(8)^ The time gap reduction between formulating ideas and publication may enable the acceleration of public policies and access to better evidence for consumers, researchers, clinicians, and decision-makers in the future. Despite all the facilities and advantages listed, there is a real concern about its interference in the authors’ creativity and a dependency relationship with ChatGPT.^(9,10)^ Initiatives such as extensions to guidelines and protocols for randomized studies related to AI, such as the ‘Consolidated Standards of Reporting Trials (CONSORT-AI)’ and the ‘Standard Protocol Items: Recommendations for Interventional Trials (SPIRIT-AI)’ extension, were the first steps to ensure regulation of its use in the scientific field.^(11,12)^ Contrarily, many efforts have made the first regulatory document published by the Secretary of State for Science, Innovation and Technology in the United Kingdom possible.^(13)^ However, the use of ChatGPT for scientific writing still requires regulation and transparency of how and when it should be used in manuscripts to enable readers the autonomy to interpret and evaluate evidence. Some organizations are publishing recommendations on AI such as ChatGPT, for academic purposes and publications.^(14,15)^ Therefore, certain aspects should be considered when authors plan and report manuscripts. The first concerns authorship and the fact that chatbots, such as ChatGPT, cannot be considered authors because of the impossibility of assuming responsibility for content to guarantee the precision and integrity of papers. In addition, they cannot understand conflicts of interest or hold copyrights.^(15,16)^ Authors’ responsibility for transparency is essential when conducting and publishing content based on ethical principles. Despite this, concerns have been raised regarding the importance of defining the admissible proportion of ChatGPT collaborations in scientific texts.^(17,18)^ Therefore, to improve transparency and data security in papers, it is important to inform the use of such tools.^(15)^

ChatGPT certainly does not replace researchers in evidence synthesis. Nevertheless, it will likely be able to increasingly help improve texts by making them more objective, attractive, and accessible, reducing language barriers, and amplifying the dissemination of evidence.^(19)^ Therefore, we, more than ever, need humans with critical evaluation skills and ethical research principles to conduct transparent research, from conception to publication, and to operate ChatGPT conscientiously and rigorously. There will be many opportunities to use ChatGPT in future as we begin to explore the benefits, controversies, and dilemmas in the coming months. Note that this letter was written only by humans!

## References

[1] Subbiah V (2023). The next generation of evidence-based medicine. Nat Med.

[2] ChatGPT OpenAI. Introducing (2023). https://openai.com/blog/chatgpt/.

[3] Bosbach WA, Senge JF, Nemeth B, Omar SH, Mitrakovic M, Beisbart C (2023). Ability of ChatGPT to generate competent radiology reports for distal radius fracture by use of RSNA template items and integrated AO classifier. Curr Probl Diagn Radiol.

[4] Ahmed SK (2023). The impact of ChatGPT on the nursing profession: revolutionizing patient care and education. Ann Biomed Eng.

[5] Babl FE, Babl MP (2023). Generative artificial intelligence: can ChatGPT write a quality abstract?. Emerg Med Australas.

[6] Kim SG (2023). Using ChatGPT for language editing in scientific articles. Maxillofac Plast Reconstr Surg.

[7] Temsah O, Khan SA, Chaiah Y, Senjab A, Alhasan K, Jamal A (2023). Overview of early ChatGPT’s presence in medical literature: insights from a hybrid literature review by ChatGPT and human experts. Cureus.

[8] Salvagno M, Taccone FS, Gerli AG (2023). Can artificial intelligence help for scientific writing?. Crit Care.

[9] Gao CA, Howard FM, Markov NS, Dyer EC, Ramesh S, Luo Y (2023). Comparing scientific abstracts generated by ChatGPT to real abstracts with detectors and blinded human reviewers. NPJ Digit Med.

[10] Flanagin A, Bibbins-Domingo K, Berkwits M, Christiansen SL (2023). Nonhuman “Authors” and implications for the integrity of scientific publication and medical knowledge. JAMA.

[11] Liu X, Cruz Rivera S, Moher D, Calvert MJ, Denniston AK (2020). SPIRIT-AI and CONSORT-AI Working Group. Reporting guidelines for clinical trial reports for interventions involving artificial intelligence: the CONSORT-AI extension. Lancet Digit Health.

[12] Liu X, Rivera SC, Moher D, Calvert MJ, Denniston AK (2020). SPIRIT-AI and CONSORT-AI Working Group. Reporting guidelines for clinical trial reports for interventions involving artificial intelligence: the CONSORT-AI Extension. BMJ.

[13] Gov. United Kingdom (2023). A pro-innovation approach to AI regulation.

[14] (2023). Tools such as ChatGPT threaten transparent science; here are our ground rules for their use. Nature.

[15] Zielinski C, Winker M, Aggarwal R, Ferris L, Heinemann M, Lapeña JF (2023). Chatbots, ChatGPT, and scholarly manuscripts: WAME recommendations on ChatGPT and chatbots in relation to scholarly publications. Natl Med J India.

[16] International Committee of Medical Journal Editors (ICMJE) (2023). Defining the Role of Authors and Contributors.

[17] Májovský M, Černý M, Kasal M, Komarc M, Netuka D (2023). Artificial Intelligence Can Generate Fraudulent but Authentic-Looking Scientific Medical Articles: Pandora’s Box Has Been Opened. Med Internet Res.

[18] Teixeira da Silva JA (2023). ChatGPT: Detection in Academic Journals is Editors’ and Publishers’ Responsibilities. Ann Biomed Eng.

[19] Sallam M (2023). ChatGPT Utility in Healthcare Education, Research, and Practice: Systematic Review on the Promising Perspectives and Valid Concerns. Healthcare (Basel).

